# Breastfeeding Self-efficacy in COVID-19 Positive Postpartum Mothers in a Community Maternal Facility in South India: A Case Control Study

**DOI:** 10.4314/ejhs.v33i1.3

**Published:** 2023-01

**Authors:** Shifa Nismath, Suchetha S Rao, Soundarya Addala, S R Ravikiran, Nutan Kamath

**Affiliations:** 1 Department of Pediatrics, Kasturba Medical College, Mangalore, Manipal Academy of Higher Education, Manipal, India

**Keywords:** Breastfeeding, COVID-19, Neonate, Postpartum, Self-Efficacy

## Abstract

**Background:**

Breastfeeding experiences have altered during the COVID-19 pandemic. Breastfeeding self-efficacy is a strong determinant of the breastfeeding behaviour of women. We aimed to study breastfeeding self-efficacy and assess the perceived factors for breastfeeding hindrance in COVID-19 positive mothers in the postpartum period.

**Method:**

A facility based case-control study was conducted with 63 COVID-19 positive (cases) and 63 COVID-19 negative postnatal mothers (controls). A breastfeeding self-efficacy short form (BFSE SF) instrument measured Breastfeeding self-efficacy 24 to 48 hours post-delivery. Mothers who tested positive for COVID-19 were interviewed about perceived factors for breastfeeding hindrance. Data was analyzed by SPSS version 25. Descriptive statistics were used for maternal parameters. BFSE SF scores were compared by a t test.

**Results:**

The mean BFSE SF score of COVID-19 positive mothers was 53.14 which was significantly lower than the mean BFSE SF score of 56.52 of COVID-19 negative mothers (p=0.013). Mothers who had received postpartum breastfeeding advice had significantly higher BFSE SF mean scores (p= 0.031). Sixty-seven percentage of COVID-19 positive mothers reported fear of transmission of illness to the neonate as a hindering factor.

**Conclusions:**

Breastfeeding self-efficacy scores were significantly lower in COVID- 19 positive mothers. Higher breastfeeding self-efficacy scores were observed in mothers who had received postpartum breastfeeding advice. The fear of transmission of the COVID-19 illness to the neonate was perceived as a breastfeeding hindering factor in most of the mothers. These observations imply the need for professional lactation support programs.

## Introduction

Exclusive breastfeeding is recommended for the first six months of an infant's life (1). Breast milk contains vital nutrients that are required for a child's growth and development, prevention of obesity, reduction in the risk of allergies, and endocrine diseases, protection from gastrointestinal and respiratory infections, promotion of mental health and psychomotor development (1). Breastfeeding promotion, practice, and support are crucial for achieving the United Nations sustainable development goals 2 and 3 to improve nutrition and secure healthy lives for all by 2030 (2).

Coronavirus disease 2019 (COVID-19) has ravaged the state of the world's economy and health. It is believed that the global pandemic may have affected maternal health, intrauterine growth, and the postnatal growth of babies. It had disrupted the continuum of care and challenged the resilience of even the most effective health systems (3). COVID-19 pandemic has affected women's breastfeeding practices. Some mothers stopped breastfeeding before they were ready due to the impact of the pandemic (4). Studies evaluating breastfeeding experiences in COVID-19 positive mothers are limited in the Indian Subcontinent.

Breastfeeding Self-Efficacy (BFSE) is a strong determinant of the breastfeeding behavior of women (5). Breastfeeding Self-Efficacy Scale Short Form (BSEF-SF) was developed by Dennis and Faux as a self-report instrument to measure maternal confidence in breastfeeding (6). Psychometric results suggest the BSES-SF is a good measure of breastfeeding self-efficacy (7). Among the various instruments used for breastfeeding self-efficacy, BFSE SF has shown strong construct validity and has been used worldwide to predict the length of breastfeeding (8, 9).

Understanding breastfeeding self-efficacy in COVID-19 positive mothers will aid in planning support for exclusive breastfeeding in future pandemics. Hence, we aimed to study breastfeeding self-efficacy by BFSE SF to assess the perceived factors for breastfeeding hindrance in COVID-19 positive mothers in the postpartum period.

## Methods

**Study design, period, and setting**: A facility-based case-control study was conducted with COVID-19 positive postnatal mothers as cases and COVID -19 negative postnatal mothers as controls from 25 June to 25 September 2021 at Government Lady Goschen Hospital, Mangalore, Karnataka, India. This hospital, located in the Coastal belt of South India, is one of the oldest hospitals in the district dating back to 1848. This hospital specializes in maternity and neonatal intensive care services with an annual delivery of around 6000 neonates. This facility serves the people of the district as well as people of border villages of neighboring states and is affiliated with Kasturba Medical College Mangalore, Manipal Academy of Higher Education, Manipal, India.

**Study population**: All the mothers who delivered at Lady Goschen Hospital during the study period were a source of the population. During the study period, hospital guidelines mandated COVID-19 testing for all pregnant women before delivery. Mothers diagnosed with COVID -19 infection by reverse transcription-polymerase chain reaction (RT- PCR) for COVID -19 within14 days before delivery were enrolled as cases, and mothers who tested negative for COVID -19 infection by RT-PCR were enrolled as controls after fulfilling inclusion and exclusion criteria. The two groups were matching in terms of other variables. A convenient sampling method was adopted for patient recruitment.

**Inclusion and exclusion criteria**: Postnatal mothers' literate in the local language Kannada, delivered at term were included. Mothers aged < 18 years and > 35 years, on drugs contraindicated for breastfeeding, not willing to breastfeed were excluded. Mothers with psychiatric illness, retroviral disease, high-risk pregnancy and postpartum complications requiring ICU admission were excluded. Neonates requiring neonatal intensive care admission and those with orofacial anomalies were excluded.

**Sample size determination**: The sample size was determined using the population mean and standard deviation formula. We used data from a previous study that showed a mean BFSE SF score of 54.8 and a standard deviation of 8.91 (10). With desired statistical power of 80%, a confidence interval of 95%, and a 5% level of significance, the sample size was calculated to be 63 for each group.

**Data collection tool and procedure**: The data collection tools included a pretested questionnaire and BFSE SF instrument. The first part of the questionnaire addressed the maternal socio-demographic details such as age, educational qualification, employment type, and family type. The second part of the questionnaire included obstetric details like antenatal visit number and place, antenatal advice on breastfeeding, parity, and previous breastfeeding experience, place of delivery, mode of delivery, gender, and birth weight of neonates. The third part of the questionnaire applied only to the COVID-19 positive group of mothers. The third part of the questionnaire included symptoms related to COVID-19 illness, if a mother received healthcare professionals' advice on precautions for nursing the baby. It also included perceived factors for breastfeeding hindrance by COVID 19 infection (COVID 19 illness-related symptoms, wearing a mask during breastfeeding, fear of transmission of COVID 19 to neonate, lack of physical presence of family members and lack of postpartum breastfeeding advice by health care professionals). In addition, there was a question that asked if breastfeeding was hindered by COVID-19 with responses on the Likert scale; 1 strongly agree, 2 agree, 3 neutral, 4 disagree, and 5 strongly disagree.

BFSE SF is a self-report instrument with 14 items to measure maternal confidence in breastfeeding. All items are assessed using a 5-point Likert-type scale where: 1 indicates not at all confident and 5 indicates always confident. This BFSE SF instrument was translated to the local language Kannada, and back translated to English by two independent language experts to check its consistency. Pediatric residents interviewed mothers in both groups 24 to 48 hours post-delivery to gather data and administered the BFSE SF Kannada version self-report instrument.

**Data quality control**: Pediatric residents involved in data collection were trained regarding the study objectives and data collection methodology. The questionnaire was pretested and suitable modification was done before the study. Interviewers were available to address any query for any item of the self-report BFSE SF instrument. Translation of BFSE SF into the local language and its consistency checking by two language experts ensured proper data. The principal investigator supervised the data collection and data entry.

**Study variables**: Breastfeeding self-efficacy of postnatal mothers measured by BFSE SF scores was a dependent variable. COVID-19 positive or negative status of the postnatal mothers was an independent variable. Further, in COVID -19 positive mothers, postnatal advice by a health professional on precautions to nurse the baby was an independent variable for breastfeeding self-efficacy.

**Data analysis**: The collected data was entered on the Statistical Package for IBM (SPSS) statistics for windows version 25.0. Armonk, NY: IBM Corp. Data were expressed as mean (standard deviation) and proportions. Student t-test, chi-square, or Fischer exact tests were used to compare data between the groups. Spearman correlation test was used to check the correlation between perception scores for breastfeeding hindrance and breastfeeding self-efficacy scores. A p-value of less than 0.05 was considered statistically significant.

**The following operational definition** was used for COVID-19 positive mothers: Mothers diagnosed with COVID -19 by RT PCR for COVID -19 within 14 days before delivery.

**Ethical approval and permissions**: Approval from the institutional ethics committee of Kasturba Medical College Mangalore was obtained (IEC KMC MLR 06-2021/192). Permission from the medical superintendent of the hospital was obtained. A patient information sheet was given, the purpose of the study was explained and patients were enrolled after informed consent. Participation in this study was voluntary. Complete anonymity of the research participants and data was maintained.

## Results

**Socio-demographic characters**: The study enrolled 63 COVID-19 positive postnatal mothers as cases and 63 COVID-19 negative postnatal mothers as controls. The mean age group of the study population was between 26 to 28 years. Most of the women in both groups had primary or secondary education. Almost 50% of mothers in both groups were homemakers. Nuclear families were more common. The socio-demographic details were comparable in both groups (Table 1).

**Table 1:** Comparison of demographic and reproductive health parameters among COVID -19 positive and COVID- 19 negative mothers

Demographic Details	COVID-19 positive mothers n=63	COVID-19 negative mothers n=63	P value
		
	N (%)	N (%)	
Age, Mean (SD)	27.84 (5.1)	26.75 (3.9)	0.179
Education			
No formal education	4 (6.3)	2 (3.2)	0.42
Primary Education	13 (20.6)	20 (31.7)	
Secondary Education	29 (46.0)	29 (46.0)	
Graduate	16 (25.4)	10 (15.9)	
Postgraduate	1 (1.6)	2 (3.2)	
Job n (%)			
Homemaker	34(53.9)	33(52.4)	0.151
Labor	13(20.6)	8(12.7)	
Beedi Roller	2(3.2)	9(14.3)	
Government Employee	7(11.1)	4(6.3)	
Private employee	7(11.1)	9(14.3)	
Family Type n (%)			
Nuclear	38(60.3)	36(57.1)	0.72
Joint	25(39.7)	27(42.9)	
Number of children n(%)			
1	26 (41.3)	23(36.5)	0.62
2	22(34.9)	20(31.7)	
≥3	15(23.8)	20(31.7)	
Previous exclusive breastfeeding n (%)			
Yes	30(47.6)	36(57.1)	0.46
No	7(11.1)	4(6.3)	
Not applicable	26(41.3)	23(36.5)	
ANC visit n (%)			
Yes	63(100)	63(100)	
No	0	0	
Breastfeeding Advice During ANC n (%)			
Yes	61(96.8)	60(95.2)	1
No	2(3.2)	3(4.8)	
Mode Of Delivery n (%)			
Vaginal	27(42.9)	31(49.2)	0.48
LSCS	36(57.1)	32(50.8)	
Gender of Neonate n (%)			
Male	28(44.4)	34(54.0)	0.29
Female	35(55.6)	29(46.0)	
Birth weight mean (SD)	2.84(0.5)	2.85(0.5)	0.96
Birth weight category (%)			
AGA	52(82.5)	51(81.0)	0.82
SGA	11(17.5)	12(19.0)	

ANC= Antenatal care; LSCS=Lower segment caesarean section; AGA=Appropriate for gestational age; SGA= Small for gestational age

**Obstetric characters of the participants and neonatal characteristics**: All study participants had antenatal checkups. The majority of the mothers had received antenatal advice on breastfeeding. Obstetric parameters were similar in COVID-19 positive and negative groups of mothers (Table 1). Twenty-three (18.3%) neonates were small for gestational age (SGA) in the study population in both cases and control groups. The proportion of SGA babies in cases and controls was comparable (Table 1).

**Breastfeeding self-efficacy scores**: BFSE SF determined Breastfeeding self-efficacy scores. The total BFSE SF scores and scores for individual questions numbered 6, 8, 9, and 12 were significantly lower in COVID-positive mothers (Table 2).

**Table 2:** Comparison of breast feeding self-efficacy scores between COVID -19 positive and COVID -19 negative mothers

	BFSE SF items	COVID-19 positive mothers (n=63) Mean (SD)	COVID-19 negative mothers (n=63) Mean (SD)	p value
1	I can always determine that my baby is getting enough milk	3.68(0.89)	3.81(0.71)	0.381
2	Successfully cope with breastfeeding like I have with other challenging tasks	3.65(0.84)	3.81(0.75)	0.270
3	Breastfeed my baby without using formula as a supplement	3.90(0.79)	4.02(0.66)	0.396
4	Ensure that my baby is properly latched on for the whole of feeding	3.98(0.88)	4.00(0.59)	0.906
5	Manage the breastfeeding situation to my satisfaction	3.87(0.90)	3.95(0.77)	0.598
6	Manage to breastfeed even if my baby is crying	3.76(0.90)	4.10(0.64)	0.017
7	Keep wanting to breastfeed	3.66(1.00)	4.19(0.53)	<0.001
8	Comfortably breastfeed with my family members present	3.62(1.05)	4.05(0.75)	0.010
9	Be satisfied with my breastfeeding experience	3.74(1.01)	4.10(0.56)	0.016
10	Deal with the fact that breastfeeding can be time consuming	3.84(1.09)	4.16(0.70)	0.055
11	Finish feeding my baby on one breast before switching to the other breast.	3.83(0.97)	4.06(0.66)	0.113
12	Continue to breastfeed my baby for every feeding.	3.76(1.07)	4.13(0.66)	0.023
13	Manage to keep up with my baby's breastfeeding demands.	3.94(0.89)	4.14(0.69)	0.151
14	Tell when my baby is finished breastfeeding	3.92(1.06)	4.08(0.72)	0.331
15	BFSE SF total score	53.14(7.91)	56.52(7.17)	0.013

BFSE SF, Breast feeding self-efficacy short form

Regarding the perceived factor, which may hinder breastfeeding during COVID-19 illness, 67 % of mothers reported the fear of transmission of illness to the neonate, followed by a lack of presence of family members in 46% of mothers (Table 3).

**Table 3:** Factors hindering breast-feeding as perceived by COVID 19 positive mothers (n=63).

Perceived factor for breast feeding hindrance *	COVID 19 positive mothers n (%)
COVID 19 illness related symptoms	11 (17.5)
Wearing mask during breast feeding	15 (23.8)
Fear of transmission of COVID 19 illness to neonate	42 (66.7)
Lack of physical presence of family members	29 (46%)
Lack of postpartum breast feeding advice by health care professionals	6 (9.5)
All of the above factors	3 (4.8)
None of the above factors	5 ((7.9)

* Multiple responses permitted

Perception scores measured on the Likert scale (1 strongly agree, 2 agree, 3 neutral, 4 disagree, and 5 strongly disagree) that breastfeeding is hindered by COVID-19 illness showed a positive correlation to breastfeeding self-efficacy score (rho 0.795, p-value < 0.0001). On the other hand, 85.7% of COVID-19 positive mothers had received advice from health professionals regarding precautions to be followed while nursing the baby. The mean total BFSE SF score in COVID-19 positive mothers was 53.14. Mothers were divided into two groups based on BFSE SF score above the mean and below the mean group and compared to the group of mothers who received advice from health professionals. The mothers who had received postpartum breastfeeding advice had significantly higher mean scores (p 0.031) (Table 4).

**Table 4:** Comparison of BFSE SF scores with postnatal advice regarding precautions while nursing the baby in COVID -19 positive mothers (n=63)

BFSE SF score	Received postnatal advice on precaution for nursing the baby (n)	Did not receive postnatal advice on precaution for nursing the baby (n)	p value
Above mean*	28	1	0.031
Below mean	26	8	

BFSE SF= Breast feeding self-efficacy short form

* Mean BFSE SF score of 53.14 was used to divide in to subgroups: above mean and below mean

**Figure 1:**
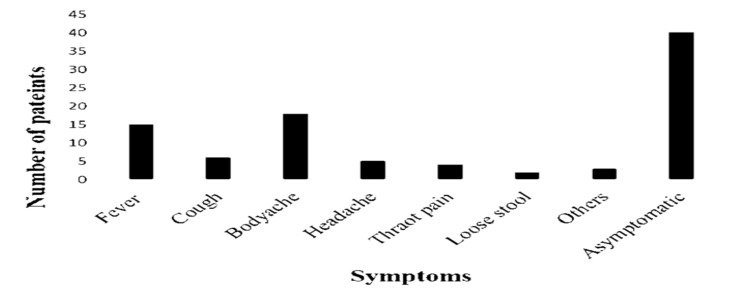
Frequency of illness related symptoms in COVID-19 positive mothers.

**Characteristics of COVID-19 positive mothers:** The mean duration of COVID-19 illness in positive mothers was 2.4 (0.7) days. Sixty-seven percentage of the COVID-positive mothers were asymptomatic. The frequency of COVID-19 related illness symptoms is shown in Figure 1.

## Discussion

COVID-19 pandemic has affected pregnant and lactating mothers directly because of the consequences of infection as well as indirectly due to restricted healthcare access, and social and economic situations (11). During the COVID-19 pandemic, increased maternal mental health problems such as anxiety and depression were reported among peri-partum women worldwide (12). Breastfeeding experiences are altered due to the COVID-19 pandemic (13).

WHO guidelines encourage mothers with suspected or confirmed COVID-19 positive illness to breastfeed as the health benefits of breastfeeding outweigh the possible risk of transmission of COVID-19 (14). Studies have reported the presence of IgG and IgA SARS-COV-2 antibodies in the breast milk of women who tested positive for COVID-19 which may protect the neonate from postnatal infection (15). Hence, it is essential to promote exclusive breastfeeding in COVID- 19 positive mothers.

Breastfeeding experiences are influenced by the mother's breastfeeding self-efficacy and social support (16). A study by Maleki-Saghooni et al., including 300 primiparous showed that social support had a significant relation to breastfeeding self-efficacy(17). The availability of professional lactation support is crucial for breastfeeding success (18). During the COVID-19 pandemic, lactating mothers faced several challenges (19). Limited professional support increased the distress of COVID-19-positive mothers (4).

Breastfeeding self-efficacy is an important predictor of breastfeeding duration. A study by Angelina et al., from Indonesia showed that higher exclusive breastfeeding rates has been associated with mothers with high breastfeeding self-efficacy scores during COVID- 19 pandemic (20). A study by Beheshti et al., which enrolled 300 breastfeeding mothers to assess the predictors of breastfeeding self-efficacy during the COVID-19 pandemic, concluded that postpartum support by spouse and intention to breastfeed was positively correlated with breastfeeding self-efficacy whereas depression was negatively correlated with breastfeeding self-efficacy (21). Simple, cost-effective measures like skin to skin contact, and Kangaroo mother care have shown to increase breastfeeding self-efficacy (22,23).

Studies assessing breastfeeding self-efficacy or feeding practices in COVID-19 positive mothers are limited. A multi-centric cohort study enrolling 125 COVID-19 positive mothers in neonatal intensive care units in Turkey showed that 56.8% of mothers adopted formula feeds, expressed breast milk was a choice in 36% of mothers and only 7.2% of mothers practiced exclusive breastfeeding. Isolation, the anxiety of parents and clinicians about the spread of infection, and the health status of mothers were some of the reasons attributed to low breastfeeding rates (24).

In the present study, we assessed the breastfeeding self-efficacy scores in COVID-19 positive mothers in the immediate postpartum period and found breastfeeding self-efficacy scores to be significantly lower in COVID- 19 positive mothers compared to COVID-19 negative mothers. COVID-19 illness-related symptoms were perceived as a reason for breastfeeding hindrance in 17.5% of mothers in our study. It is noteworthy that 63.5% of COVID-19 positive mothers were asymptomatic.

Sixty seven percent of mothers perceived fear of transmission of the virus to neonates as a hindering factor for breastfeeding. At the beginning of the pandemic, there was a lack of information regarding mother-to-child transmission of SARS-CoV-2 through breast milk. This led to the spread of the misconception that newborns have a higher risk of being infected with SARS-CoV-2 from positive mothers (25). Our study was conducted during the second wave of the pandemic when the safety of breastfeeding for COVID-19 positive mothers was well established. The high frequency of mothers reporting the fear of transmission to neonates as a hindering factor despite the guidelines giving information on the safety of breastfeeding is an alarming condition.

Social support can have a significant impact on a mother's ability to initiate and continue breastfeeding. COVID-19 positive mothers were negatively impacted due to a lack of in-person support from family, peers, and lactation counselors (26). In the present study, 46 % of mothers perceived being away from family members as a hindering factor for breastfeeding.

Postnatal breastfeeding advice by health care professionals was received in 85.7 % of COVID-19 positive mothers in this study. Breastfeeding self-efficacy scores above the mean were significantly associated with postpartum breastfeeding advice (p = 0.031). A study from Indonesia reported early initiation of breast feeding following mobile -health interactive messages during the COVID-19 pandemic (27). Such strategies may be adopted for COVID-19 positive mothers when in person lactation support may be challenging.

To conclude, breastfeeding self-efficacy scores were significantly lower in COVID-19 positive mothers. Higher breastfeeding self-efficacy scores were observed in COVID-19 positive mothers who received postpartum breastfeeding advice. Fear of transmission of COVID-19 to the neonate was the highest reported breastfeeding hindering factor. These observations imply the need for professional lactation support programs for infected postpartum women during future pandemics. Multi-centric cohort studies with larger sample sizes may give better direction on the needs of mothers of various socioeconomic strata and educational backgrounds to aid in designing lactation support programs.
